# Comparison of reader agreement, correlation with liver biopsy, and time-burden sampling strategies for liver proton density fat fraction measured using magnetic resonance imaging in patients with obesity: a secondary cross-sectional study

**DOI:** 10.1186/s12880-022-00821-6

**Published:** 2022-05-17

**Authors:** Di Cao, Mengyi Li, Yang Liu, He Jin, Dawei Yang, Hui Xu, Han Lv, JIa Liu, Peng Zhang, Zhongtao Zhang, Zhenghan Yang

**Affiliations:** 1 Department of Radiology, Beijing Friendship Hospital, Capital Medical University, No. 95 Yong-an Road, Xi-Cheng District, Beijing, 100050 China; 2 Department of General Surgery, Beijing Friendship Hospital, Capital Medical University and National Clinical Research Center for Digestive Diseases, No. 95 Yong-an Road, Xi-Cheng District, Beijing, 100050 China

**Keywords:** Liver biopsy, Magnetic resonance imaging (MRI)-based proton density fat fraction (MRI-PDFF), Region-of-interest

## Abstract

**Background:**

The magnetic resonance imaging (MRI)-based proton density fat fraction (PDFF) has become popular for quantifying liver fat content. However, the variability of the region-of-interest (ROI) sampling strategy may result in a lack of standardisation of this technology. In an effort to establish an accurate and effective PDFF measurement scheme, this study assessed the pathological correlation, the reader agreement, and time-burden of different sampling strategies with variable ROI size, location, and number.

**Methods:**

Six-echo spoiled gradient-recalled-echo magnitude-based fat quantification was performed for 50 patients with obesity, using a 3.0-T MRI scanner. Two readers used different ROI sampling strategies to measure liver PDFF, three times. Intra-reader and inter-reader agreement was evaluated using intra-class correlation coefficients and Bland‒Altman analysis. Pearson correlations were used to assess the correlation between PDFFs and liver biopsy. Time-burden was recorded.

**Results:**

For pathological correlations, the correlations for the strategy of using three large ROIs in Couinaud segment 3 (S3 3L-ROI) were significantly greater than those for all sampling strategies at the whole-liver level (*P* < 0.05). For inter-reader agreement, the sampling strategies at the segmental level for S3 3L-ROI and using three large ROIs in Couinaud segment 6 (S6 3L-ROI) and the sampling strategies at the whole-liver level for three small ROIs per Couinaud segment (27S-ROI), one large ROI per Couinaud segment (9L-ROI), and three large ROIs per Couinaud segment (27S-ROI) had limits of agreement (LOA) < 1.5%. For intra-reader agreement, the sampling strategies at the whole-liver level for 27S-ROI, 9L-ROI, and 27L-ROI had both intraclass coefficients > 0.995 and LOAs < 1.5%. The change in the time-burden was the largest (100.80 s) when 9L-ROI was changed to 27L-ROI.

**Conclusions:**

For hepatic PDFF measurement without liver puncture biopsy as the gold standard, and for general hepatic PDFF assessment, 9L-ROI sampling strategy at the whole-liver level should be used preferentially. For hepatic PDFF with liver puncture biopsy as the gold standard, 3L-ROI sampling strategy at the puncture site segment is recommended.

## Background

Nonalcoholic fatty liver disease (NAFLD) involves the excessive deposition of triglycerides in the liver and has become a major public health concern globally [1]. Obesity is an important risk factor for NAFLD and nonalcoholic steatohepatitis (NASH) [2, 3]. Without intervention, fatty liver may develop into steatohepatitis, liver fibrosis, liver cirrhosis, and hepatocellular carcinoma [1, 4, 5]. Recently, a consensus of international experts has proposed a change in the disease name from NAFLD to metabolic-associated fatty liver disease (MAFLD), which is a positive diagnosis that reflects the close relationship between fatty liver and overnutrition, sedentary lifestyle, and metabolic conditions [6]. The criteria are based on evidence of hepatic steatosis, plus any of the following three conditions: Overweight/obesity, presence of type 2 diabetes mellitus, or presence of metabolic disorder [7].

Currently, the gold standard for the diagnosis and grading of hepatic steatosis is liver biopsy. However, this procedure can occasionally lead to complications, such as bleeding, liver hematoma, and puncture of the gallbladder or adjacent organs [8–12]. For these reasons, noninvasive detection might be the preferred method for detecting MAFLD/NAFLD.

Over the past 20 years, there has been a shift from qualitative to quantitative assessment of liver steatosis by imaging, with current research investigating the use of ultrasound, computed tomography, and magnetic resonance imaging (MRI) for improving the accuracy of fat quantification [13–15]. MRI-based proton density fat fraction (MRI-PDFF) with a multi-echo chemical-shift-encoded sequence is now routinely used at different centres to quantify hepatic steatosis, with excellent diagnostic performance [13–18]. This assessment utilises multi-echo water‒lipid separation technology to eliminate the interference of T1 and T2* effects, as well as main magnetic field (B0) inhomogeneity in the fat quantification process, which makes accurate quantification of visceral fat possible [19].

The optimal sampling strategy based on the regions-of-interest (ROIs) has not yet been established. The PDFF of the liver is usually evaluated by radiologists by manually placing different numbers of ROIs at different locations of the liver and averaging these values [16–22]. Furthermore, the spatial distribution of liver fat is not uniform, which results in different PDFFs at different locations [22–24]. This lack of standardisation leads to further PDFF variability, which affects the widespread use of this technology as a quantitative imaging biomarker. Currently, the common approach in clinical trials is to place 1–3 ROIs from each Couinaud liver segment [22, 23, 25, 26]. Several reports have indicated that large ROIs with a 4-ROI (anterior, posterior, medial, and lateral), balanced 4-ROI (2 ROIs per lobe), 9-ROI (1 ROI per Couinaud segment), or 27-ROI strategy (3 ROIs per Couinaud segment) are preferred for good intra- and inter-reader agreements [20, 21, 24, 26–29]. The common sampling method measures PDFF at the whole-liver level, which requires a longer time [28, 29]. Some studies have suggested the use of a balanced 4-ROI (2 ROIs per lobe) approach, which provides high repeatability and low error, in order to reduce time consumption [20, 21]. Although the above multi-ROI method provide good reader agreements, which can avoid the variability of sampling, the gold standard for detecting "steatosis" is liver biopsy, which can accurately reflect the histological situation of the liver at the puncture site. Changing of ROI sampling strategies may vary in performance in terms of pathological correlation between liver biopsy and PDFF.

Therefore, in an effort to establish an accurate and effective PDFF measurement scheme for clinicians and radiologists, this study assessed the pathological correlation, the intra- and inter-reader agreement, and time-burden of different sampling strategies with variable ROI size, location, and number.

## Methods

### Study design and patients

We performed a secondary analysis of cross-sectional data from a prospective, self-controlled observational study conducted by Li et al. [26] at the Beijing Friendship Hospital, Beijing, China, from December 2017 to November 2018. The study used MRI-PDFF to determine the changes in hepatic PDFF characteristics after bariatric surgery, to determine the correlation with the clinical NASH score [30], and to determine the predictors of postoperative NASH score changes. The inclusion criteria were an age between 18 and 65 years, diagnosis of obesity (BMI ≥ 28 kg/m^2^) [31, 32], and willingness to accept bariatric surgery intervention. The exclusion criteria were a history of obesity surgery interventions or general contraindications for MRI.

Sixty-nine patients were enrolled and underwent abdominal MRI at baseline and again at 3 months after surgery. Fifty patients underwent liver biopsy at baseline. We used the pathological biopsy as the pathological reference standard of these 50 patients and measured their liver PDFFs at baseline. Eleven of 50 patients had a history of type 2 diabetes for at least half a year and regularly used insulin, metformin, or other hypoglycaemic drugs. Standard clinical, anthropometric, and biochemical measurements of these 50 patients at baseline were also obtained. The parameters included sex, age, body weight, body mass index (BMI), and serum levels of alanine aminotransferase, alanine aminotransferase, triglycerides, and glycated haemoglobin.

The study was approved by the Ethics Committees of the Beijing Friendship Hospital where the study was conducted (NO. 2017-P2-131–02). Informed consent was obtained from the patients for the use and publication of their data.

### Liver biopsy and histopathological analysis

According to the international surgical standards, all patients underwent laparoscopic Roux-en-Y gastric shunt or sleeve gastrectomy bariatric surgery. Wedge liver biopsy was performed in the left liver lobe (area of Segment 3) at the same time as the surgery, by a single operator.

Liver biopsy material were fixed in 10% buffered formalin, and was sent to the Biobank of Beijing Friendship Hospital for unified pathological sectioning and staining (haematoxylin‒eosin, reticular fibre, Masson, copper, and iron staining). Haematoxylin‒eosin staining was used to evaluate hepatic steatosis, as the pathological reference standard. A single liver pathologist with 12 years of experience, who was blinded to the clinical and radiological data, used the near continuous scale (0%, 5%, 10%, 20%, 30%, …, 100%) to score hepatic steatosis. The mean value of the fatty degeneration score was recorded and converted to the following four-point ordinal score, as defined by the NASH Clinical Research Network scoring system [33]: 0 (< 5% hepatocytes), 1 (5–33% hepatocytes), 2 (33–66% hepatocytes), and 3 (> 66% hepatocytes).

### MRI quantification technique

MRI was performed within 1‒2 days before surgery. All patients underwent non-contrast scans using a 3.0-Tesla MRI scanner (MR750, GE Healthcare, Waukesha, WI, USA) with an eight-channel phased-array body coil centred over the liver. The patients were instructed to fast for 6 h before undergoing scanning with feet first in the supine position. A six-echo spoiled gradient-recalled-echo magnitude-based fat quantification technique was used. The parameters were as follows: repetition time, 7.3 ms; echo time, six different echoes ranging from 1.0 to 5.0 ms to permit correction for R2* signal decay and chemical-shift-based separation of fat and water signals; matrix, 160 × 160; field-of-vision, 40 cm; slice-section thickness, 8 mm; number of excitations, 0.5; flip angle, low (4°) to minimise T1 bias [34]; bandwidth, 111.11; and acquisition time, 21 s. Respiratory bellows were applied to monitor breathing, and the patients were instructed to hold their breath during image acquisition. The sequences were planned such that the whole liver was covered.

### PDFF measurements

A radiologist with 8 years of experience in abdominal imaging diagnosis (Reader 1) and another with 3 years of experience in abdominal imaging diagnosis (Reader 2) used the Picture Archiving and Communication System (PACS) to view the images.

To verify the impact of different ROI sampling strategies (different location, number, and size) on intra- and inter-reader agreement, pathological correlation, and time-burden, we used ROIs at different locations, different numbers, and different sizes for analysis according to the sampling strategies reported in previous studies [20, 21, 24, 26–29]. Locations included each of the Couinaud segments of the liver at the segmental level (including segment 1 [S1], segment 2 [S2], segment 3 [S3], segment 4a [S4a], segment 4b [S4b], segment 5 [S5], segment 6 [S6], segment 7 [S7] and segment 8 [S8]), as well as two points in the left and right lobes and all nine Couinaud segments at the whole liver level. Readers freely selected the slices. For quantity, first, each reader manually placed one and three ROIs in each of the nine Couinaud segments, and then two ROIs in the left and right lobes. Therefore, the number of ROIs included: one ROI (one ROI in a single liver segment), three ROIs (three ROIs in a single liver segment), nine ROIs (one ROI in each Couinaud segment), 27 ROIs (three ROIs in each Couinaud segment), and four ROIs (two ROIs in the left lobe, two ROIs in the right lobe). In terms of size, we used large (> 4 cm^2^; if the S1 volume was small, we used the largest area that fit inside each placement to make ROIs > 3 cm^2^) and small (< 4 cm^2^; if the S1 volume was small, we used an ROI < 3 cm^2^) ROIs to measure PDFF. Large vessels, hepatic margin, and obvious image artifacts were avoided.

**Fig. 1:**
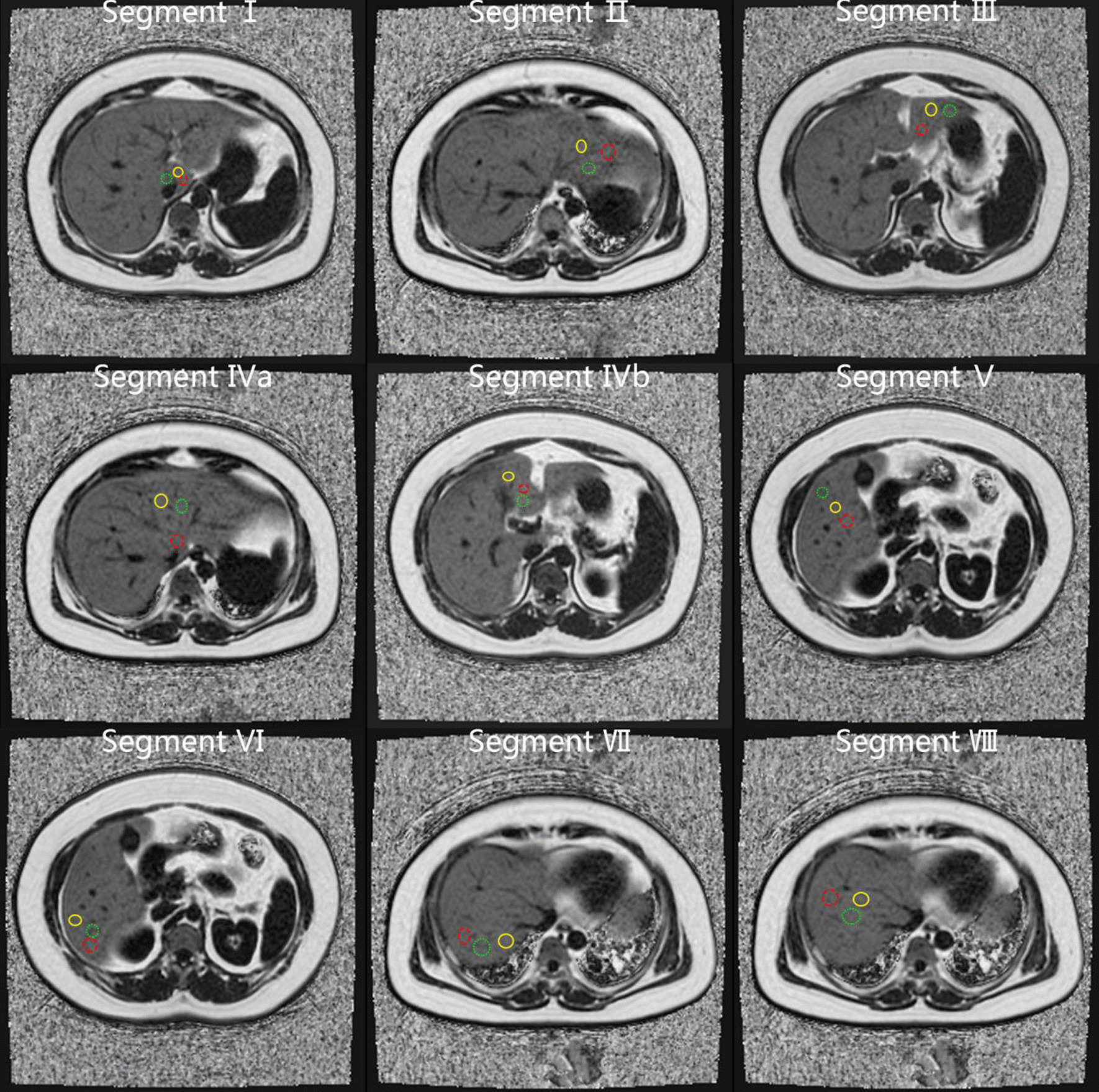
Visual representation of sampling strategy of the location and number of regions-of-interest (ROIs) placed by the readers on proton density fat fraction (PDFF) maps. Yellow, red, and green circles represent the first, second, and third ROIs placed in each liver segment, respectively. The PDFF measured by yellow ROIs on each single liver segment represents 1S-ROI. The mean PDFF measured by yellow, red, and green ROIs on each single liver segment represents 3S-ROI. The mean PDFF measured by all yellow ROIs on all liver segments represents 9S-ROI. The mean PDFF measured by all yellow, red, and green ROIs on all liver segments represents 27S-ROI

**Fig. 2:**
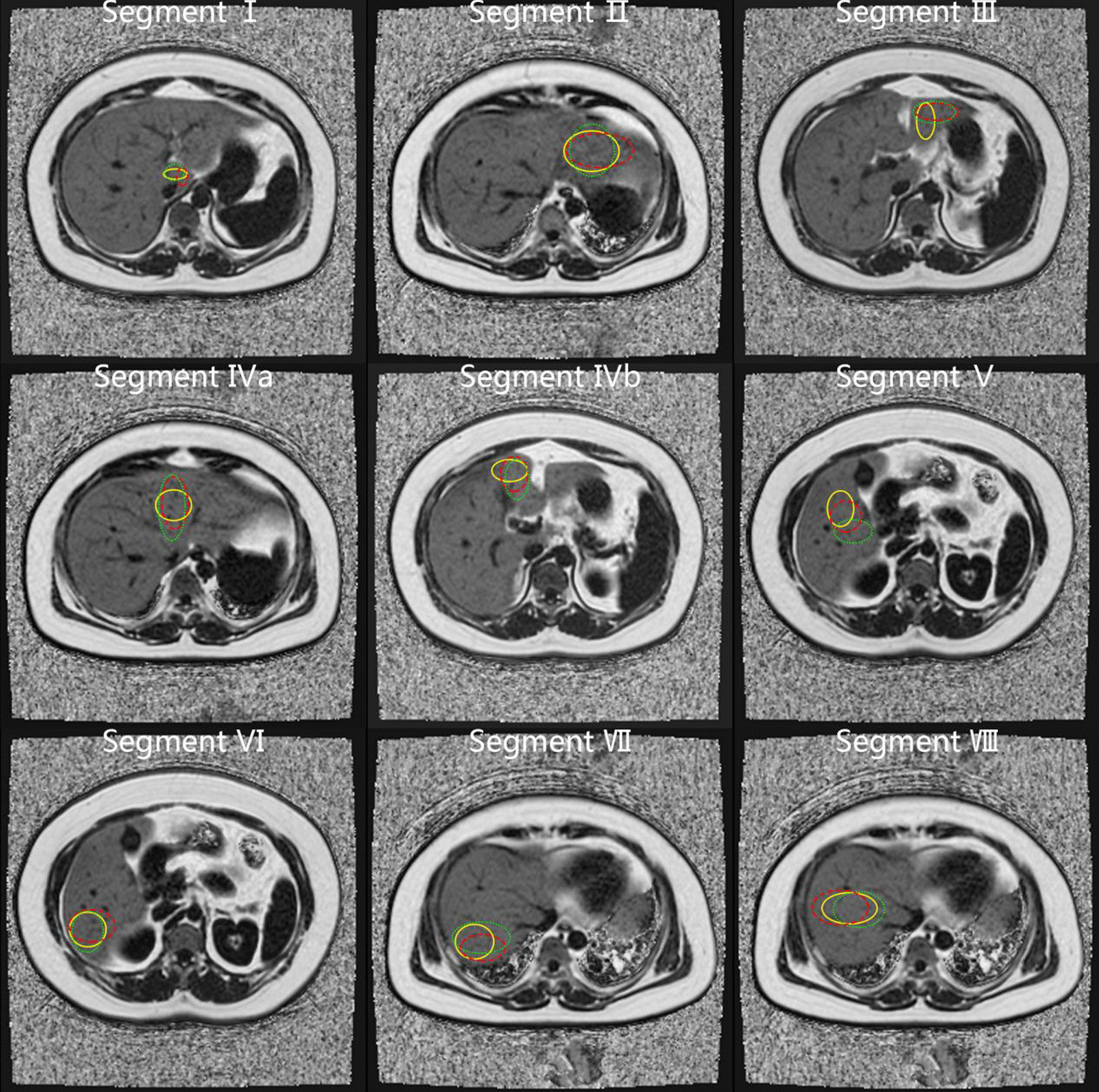
Visual representation of sampling strategy of the location and number of large regions-of-interest (ROIs) placed by the readers on proton density fat fraction (PDFF) maps; yellow, red, and green circles represent the first, second, and third ROIs placed in each liver segment, respectively. The PDFF measured by yellow ROIs on each single liver segment represents 1L-ROI. The mean PDFF measured by yellow, red, and green ROIs on each single liver segment represents 3L-ROI. The mean PDFF measured by all yellow ROIs on all liver segments represents 9L-ROI. The mean PDFF measured by all yellow, red, and green ROIs on all liver segments represents 27L-ROI

**Fig. 3:**
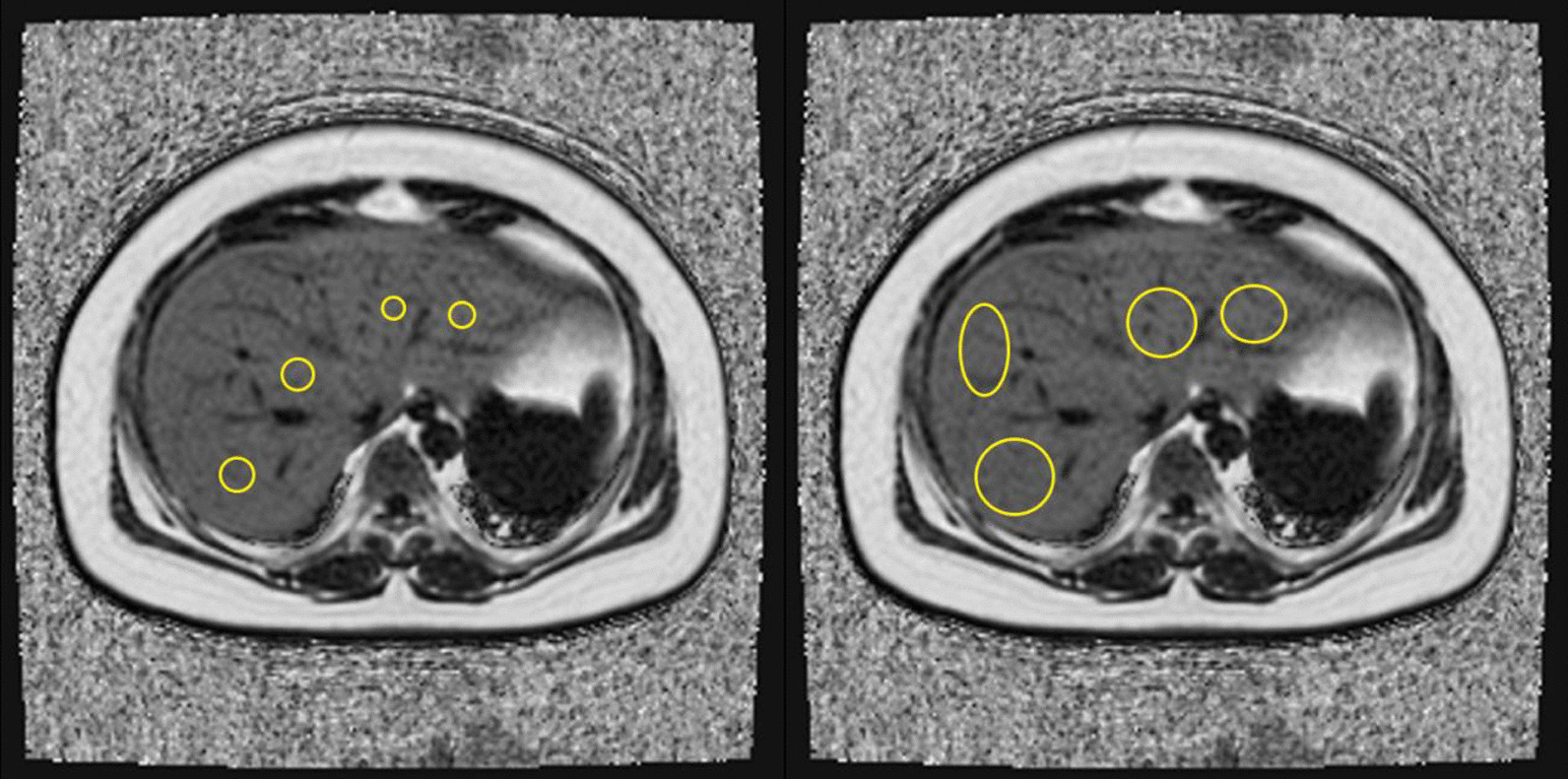
Visual representation of the sampling strategies of 4S region-of-interest (ROI) and 4L-ROI (yellow circle) at the whole-liver level placed by the readers on proton density fat fraction maps

Through the above methods, we achieved different sampling strategies as follows: one small ROI in a single Couinaud segment (1S-ROI strategy) at the segmental level, three small ROIs in a single Couinaud segment (3S-ROI strategy) at the segmental level, one large ROI in a single Couinaud segment (1L-ROI strategy) at the segmental level, three large ROIs in a single Couinaud segment (3L-ROI strategy) at the segmental level, one small ROI per Couinaud segment (9S-ROI strategy) at the whole-liver level, three small ROIs per Couinaud segment (27S-ROI strategy) at the whole-liver level, one large ROI per Couinaud segment (9L-ROI strategy) at the whole-liver level, three large ROIs per Couinaud segment (27S-ROI strategy) at the whole-liver level, two small ROIs in the left and right lobes (4S-ROI strategy), and two large ROIs in the left and right lobes (4L-ROI strategy) at the whole-liver level (Figs. 1, 2, 3).

Three months later, Reader 1 re-measured the PDFFs in the original image to assess the intra-reader agreement, using the same measurement method as that used the first time. Consequently, we obtained three measurement results for two readers.

The MRI-PDFF images were assessed by readers in a double-blind method at the image analysis. However, Reader 1 knew that the image of the second measurement was the same as that used in the first round. The two readers were also blinded to the clinical data and the histological data.

### Time-burden

We recorded the time-burden for one person (Reader 1, first time) to measure the time for each sampling strategy. The recorded time encompassed opening the PDFF diagram in the PACS, selecting the slice, drawing the ROI on the PDFF diagram, and exporting the ROI measurement data to Excel (version 2016, Microsoft Corp, Redmond, WA, USA). The time-burden at the segmental level was recorded as the average of the total time spent in each Couinaud segment using one sampling strategy. The time-burden at the whole-liver level was recorded as the time spent using one sampling strategy.

### Statistical analysis

Pearson correlations were used to assess the correlation between the histological degree of steatosis and PDFF. To determine whether the pathological correlations had significant differences, we compared the pathological correlations of each sampling strategy at the segmental and whole-liver levels, and pairwise comparison was made between the pathological correlations of two sampling strategies at the whole-liver level (Tables 1, 2, 3). Because the procedure involved three measurements for time, the above correlations were compared and analysed for each time measurement dataset.

**Table 1:** Correlation between proton density fat fraction measured by different sampling strategies and liver biopsy (Reader 1, first time)

	Correlation (r)	Correlation comparison					
		4S-ROI	4L-ROI	9S-ROI	27S-ROI	9L-ROI	27L-ROI
S1 1S-ROI	0.835	−	−	−	−	−	−
S2 1S-ROI	0.822	−	−	−	−	−	−
S3 1S-ROI	0.878	−	−	−	−	−	−
S4a 1S-ROI	0.845	−	−	−	−	−	−
S4b 1S-ROI	0.803	−	−	−	−	−	−
S5 1S-ROI	0.820	−	−	−	−	−	−
S6 1S-ROI	0.829	−	−	−	−	−	−
S7 1S-ROI	0.829	−	−	−	−	−	−
S8 1S-ROI	0.831	−	−	−	−	−	−
S1 3S-ROI	0.839	−	−	−	−	−	−
S2 3S-ROI	0.848	−	−	−	−	−	−
S3 3S-ROI	0.889	−	−	+	+	−	−
S4a 3S-ROI	0.858	−	−	−	−	−	−
S4b 3S-ROI	0.844	−	−	−	−	−	−
S5 3S-ROI	0.831	−	−	−	−	−	−
S6 3S-ROI	0.825	−	−	−	−	−	−
S7 3S-ROI	0.831	−	−	−	−	−	−
S8 3S-ROI	0.860	−	−	−	−	−	−
S1 1L-ROI	0.857	−	−	−	−	−	−
S2 1L-ROI	0.849	−	−	−	−	−	−
S3 1L-ROI	0.878	−	−	−	−	−	−
Sa4 1L-ROI	0.852	−	−	−	−	−	−
S4b 1L-ROI	0.857	−	−	−	−	−	−
S5 1L-ROI	0.863	−	−	−	−	−	−
S6 1L-ROI	0.829	−	−	−	−	−	−
S7 1L-ROI	0.827	−	−	−	−	−	−
S8 1L-ROI	0.825	−	−	−	−	−	−
S1 3L-ROI	0.853	−	−	−	−	−	−
S2 3L-ROI	0.870	−	−	−	−	−	−
S3 3L-ROI	0.896	+	+	+	+	+	+
S4a 3L-ROI	0.857	−	−	−	−	−	−
S4b 3L-ROI	0.868	−	−	−	−	−	−
S5 3L-ROI	0.832	−	−	−	−	−	−
S7 3L-ROI	0.819	−	−	−	−	−	−
S6 3L-ROI	0.819	−	−	−	−	−	−
S8 3L-ROI	0.868	−	−	−	−	−	−
4S-ROI	0.864		−	−	−	−	−
4L-ROI	0.860			−	−	−	−
9S-ROI	0.857				−	−	−
27S-ROI	0.860					-	-
9L-ROI	0.866						−
27L-ROI	0.868						

(−) indicates that there is no significant difference in the comparison of correlations or that the correlation of the segmental level sampling strategy is lower than that of the whole-liver level sampling strategy

( +) indicates that there are significant differences in the comparison of correlations and that the correlation of the segmental level sampling strategy is higher than that of the whole-liver level sampling strategy

*S-ROI* Small region-of-interest, *L-ROI* Large region-of-interest

**Table 2:** Correlation between proton density fat fraction measured by different sampling strategies and liver biopsy (Reader 1, second time)

	Correlation (r)	Correlation comparison					
		4S-ROI	4L-ROI	9S-ROI	27S-ROI	9L-ROI	27L-ROI
S1 1S-ROI	0.844	−	−	−	−	−	−
S2 1S-ROI	0.822	−	−	−	−	−	−
S3 1S-ROI	0.850	−	−	−	−	−	−
S4a 1S-ROI	0.871	−	−	−	−	−	−
S4b 1S-ROI	0.835	−	−	−	−	−	−
S5 1S-ROI	0.845	−	−	−	−	−	−
S6 1S-ROI	0.825	−	−	−	−	−	−
S7 1S-ROI	0.805	−	−	−	−	−	−
S8 1S-ROI	0.820	−	−	−	−	−	−
S1 3S-ROI	0.837	−	−	−	−	−	−
S2 3S-ROI	0.865	−	−	−	−	−	−
S3 3S-ROI	0.897	+	+	+	+	-	+
S4a 3S-ROI	0.881	−	−	−	−	−	−
S4b 3S-ROI	0.876	−	−	−	−	−	−
S5 3S-ROI	0.860	−	−	−	−	−	−
S6 3S-ROI	0.826	−	−	−	−	−	−
S7 3S-ROI	0.807	−	−	−	−	−	−
S8 3S-ROI	0.825	−	−	−	−	−	−
S1 1L-ROI	0.873	−	−	−	−	−	−
S2 1L-ROI	0.876	−	−	−	−	−	−
S3 1L-ROI	0.864	−	−	−	−	−	−
Sa4 1L-ROI	0.884	−	−	−	−	−	−
S4b 1L-ROI	0.845	−	−	−	−	−	−
S5 1L-ROI	0.850	−	−	−	−	−	−
S6 1L-ROI	0.833	−	−	−	−	−	−
S7 1L-ROI	0.807	−	−	−	−	−	−
S8 1L-ROI	0.838	−	−	−	−	−	−
S1 3L-ROI	0.852	−	−	−	−	−	−
S2 3L-ROI	0.879	−	−	−	−	−	−
S3 3L-ROI	0.895	+	+	+	+	+	+
S4a 3L-ROI	0.888	−	+	−	−	−	+
S4b 3L-ROI	0.854		−	−	−	−	−
S5 3L-ROI	0.858	−	−	−	−	−	−
S7 3L-ROI	0.827	−	−	−	−	−	−
S6 3L-ROI	0.818	−	−	−	−	−	−
S8 3L-ROI	0.827	−	−	−	−	−	−
4S-ROI	0.866		−	−	−	−	−
4L-ROI	0.861			−	−	−	−
9S-ROI	0.866				-	−	−
27S-ROI	0.871					−	−
9L-ROI	0.873						−
27L-ROI	0.867						

(−) indicates that there is no significant difference in the comparison of correlations or that the correlation of the segmental level sampling strategy is lower than that of the whole-liver level sampling strategy

( +) indicates that there are significant differences in the comparison of correlations and that the correlation of the segmental level sampling strategy is higher than that of the whole-liver level sampling strategy

S-ROI, small region-of-interest; L-ROI, large region-of-interest

**Table 3:** Correlation between proton density fat fraction measured by different sampling strategies and liver biopsy (Reader 2)

	Correlation (r)	Correlation comparison					
		4S-ROI	4L-ROI	9S-ROI	27S-ROI	9L-ROI	27L-ROI
S1 1S-ROI	0.841	−	−	−	−	−	−
S2 1S-ROI	0.825	−	−	−	−	−	−
S3 1S-ROI	0.865	−	−	−	−	−	−
S4a 1S-ROI	0.881	−	−	−	−	−	−
S4b 1S-ROI	0.854	−	−	−	−	−	−
S5 1S-ROI	0.823	−	−	−	−	−	−
S6 1S-ROI	0.838	−	−	−	−	−	−
S7 1S-ROI	0.824	−	−	−	−	−	−
S8 1S-ROI	0.796	−	−	−	−	−	−
S1 3S-ROI	0.834	−	−	−	−	−	−
S2 3S-ROI	0.820	−	−	−	−	−	−
S3 3S-ROI	0.895	−	−	−	−	−	−
S4a 3S-ROI	0.877	−	−	−	−	−	−
S4b 3S-ROI	0.850	−	−	−	−	−	−
S5 3S-ROI	0.860	−	−	−	−	−	−
S6 3S-ROI	0.841	−	−	−	−	−	−
S7 3S-ROI	0.832	−	−	−	−	−	−
S8 3S-ROI	0.824	−	−	−	−	−	−
S1 1L-ROI	0.839	−	−	−	−	−	−
S2 1L-ROI	0.862	−	−	−	−	−	−
S3 1L-ROI	0.878	−	−	−	−	−	−
Sa4 1L-ROI	0.875	−	−	−	−	−	−
S4b 1L-ROI	0.859	−	−	−	−	−	−
S5 1L-ROI	0.865	−	−	−	−	−	−
S6 1L-ROI	0.825	−	−	−	−	−	−
S7 1L-ROI	0.788	−	−	−	−	−	−
S8 1L-ROI	0.851	−	−	−	−	−	−
S1 3L-ROI	0.849	−	−	−	−	−	−
S2 3L-ROI	0.873	−	−	−	−	−	−
S3 3L-ROI	0.904	+	+	+	+	+	+
S4a 3L-ROI	0.879	−	−	−	−	−	−
S4b 3L-ROI	0.855	−	−	−	−	−	−
S5 3L-ROI	0.861	−	−	−	−	−	−
S7 3L-ROI	0.821	−	−	−	−	−	−
S6 3L-ROI	0.811	−	−	−	−	−	−
S8 3L-ROI	0.852	−	−	−	−	−	−
4S-ROI	0.868		−	−	−	−	−
4L-ROI	0.875			−	−	−	−
9S-ROI	0.869				−	−	−
27S-ROI	0.867					−	−
9L-ROI	0.868						−
27L-ROI	0.872						

(−) indicates that there is no significant difference in the comparison of correlations or that the correlation of the segmental level sampling strategy is lower than that of the whole-liver level sampling strategy

( +) indicates that there are significant differences in the comparison of correlations and that the correlation of the segmental level sampling strategy is higher than that of the whole-liver level sampling strategy

*S-ROI* Small region-of-interest, *L-ROI* Large region-of-interest

Intra-reader and inter-reader agreement were evaluated using intra-class correlation coefficients (ICCs) and Bland‒Altman analyses. The consistency between the first and second PDFF measurements of Reader 1 represents the intra-reader agreement. The consistency between the first PDFF measurement of Reader 1 and PDFF measurements of Reader 2 represented the inter-reader agreement. We used two thresholds to define close agreement: ICCs > 0.995 and absolute limits of agreement (LOA) < 1.5%. These thresholds were selected a priori to represent clinically meaningful differences [20].

The pathological correlation differences were compared relied on tests implemented in the *cocor* R package [35] by calculating Fisher’s z. Other statistical analyses were performed using MedCalc v. 18.2.1 (MedCalc Software, Ostend, Belgium) and SPSS v25.0 (IBM Inc., Armonk, NY, USA). The significance threshold was set at *p* = 0.05.

## Results

### Patient characteristics

The study sample comprised 40 women (mean age: 32.85 ± 8.88 years; range, 17–54 years) and 10 men (mean age: 37.20 ± 8.19 years; range, 22–50 years). The overall average age of 50 patients was 33.72 ± 8.84 years (range, 17–54 years). The summaries of body weight, BMI, liver biochemical parameters, and blood lipid and HbA1c levels, and histological degree of steatosis in 50 patients are presented in Table 4.

**Table 4:** Participants' characteristics (*n* = 50)

Characteristic	Value (min–max)	Number of patients with values outside the normal range (normal range)
Male	10	–
Female	40	–
Mean age (years)	33.72 ± 8.84 (18–54)	–
Mean weight, kg	107.92 ± 24.11 (72.00–177.40)	–
Mean BMI, kg/m^2^	38.15 ± 6.57 (28.15–52.72)	50 (18.5–23.9)
Mean ALT, U/L	38.82 ± 39.53 (8.00–181.00)	11 (9.00–50.00)
Mean AST, U/L	26.66 ± 19.09 (11.40–94.10)	8 (15.00–40.00)
Mean TG, mmol/L	2.44 ± 2.63 (0.67–15.16)	22 (0.57–1.70)
Mean HbA1c, %	6.35 ± 1.62 (0.45–10.40)	15 (4.27–6.07)
*Steatosis grade*		
0 (< 5% hepatocytes)	5	–
1 (5–33% hepatocytes)	22	–
2 (33–66% hepatocytes)	15	–
3 (> 66% hepatocytes)	8	–

*BMI* Body mass index, *ALT* Alanine aminotransferase, *AST* Aspartate aminotransferase, *TG* Triglycerides, *HbA1c* Glycosylated haemoglobin

### PDFF measurement

Liver PDFFs measured by different strategies by Readers 1 and 2 are summarised in Table 5.

**Table 5:** PDFFs (%) of all sampling strategies measured by readers 1 and 2

	Reader 1 PDFF measure		Reader 2 PDFF measure
	First time	Second time	
S1 1S-ROI	12.02 ± 9.77	11.91 ± 9.43	11.83 ± 8.97
S2 1S-ROI	12.41 ± 8.19	12.96 ± 8.63	12.11 ± 8.39
S3 1S-ROI	13.19 ± 9.84	13.23 ± 8.80	12.02 ± 9.28
S4a 1S-ROI	13.27 ± 9.01	12.87 ± 8.98	13.35 ± 9.37
S4b 1S-ROI	13.34 ± 9.57	13.04 ± 9.85	13.41 ± 9.69
S5 1S-ROI	13.31 ± 9.35	13.54 ± 9.78	13.56 ± 9.81
S6 1S-ROI	13.95 ± 9.81	13.59 ± 9.47	12.94 ± 9.03
S7 1S-ROI	14.76 ± 9.59	14.67 ± 10.02	14.37 ± 9.61
S8 1S-ROI	14.59 ± 9.95	14.66 ± 9.25	14.63 ± 9.48
S1 3S-ROI	11.96 ± 9.54	12.04 ± 9.16	11.97 ± 8.96
S2 3S-ROI	12.57 ± 8.35	12.79 ± 8.71	13.00 ± 8.72
S3 3S-ROI	12.21 ± 9.44	13.14 ± 9.15	12.62 ± 9.11
S4a 3S-ROI	13.21 ± 9.11	12.92 ± 8.87	13.56 ± 9.81
S4b 3S-ROI	13.40 ± 9.33	13.56 ± 9.71	13.06 ± 9.43
S5 3S-ROI	13.61 ± 9.53	13.49 ± 9.54	13.43 ± 9.51
S6 3S-ROI	14.04 ± 9.89	13.28 ± 9.27	13.07 ± 8.83
S7 3S-ROI	14.73 ± 9.74	14.68 ± 9.80	14.68 ± 9.58
S8 3S-ROI	14.50 ± 9.74	14.74 ± 9.24	14.61 ± 9.46
S1 1L-ROI	11.84 ± 9.12	11.76 ± 8.92	11.53 ± 9.58
S2 1L-ROI	12.12 ± 9.81	12.21 ± 8.72	12.17 ± 9.07
S3 1L-ROI	12.90 ± 9.21	13.05 ± 9.20	13.68 ± 9.26
Sa4 1L-ROI	12.56 ± 9.20	12.87 ± 9.20	12.82 ± 8.90
S4b 1L-ROI	13.50 ± 9.53	12.94 ± 9.27	13.14 ± 9.08
S5 1L-ROI	13.30 ± 9.61	13.38 ± 9.69	13.32 ± 9.07
S6 1L-ROI	13.33 ± 9.48	13.69 ± 9.68	13.45 ± 9.59
S7 1L-ROI	14.39 ± 9.90	14.39 ± 10.07	14.38 ± 9.83
S8 1L-ROI	14.06 ± 9.15	14.41 ± 9.52	14.11 ± 9.33
S1 3L-ROI	11.68 ± 9.17	11.57 ± 9.07	11.52 ± 9.33
S2 3L-ROI	12.30 ± 8.61	12.22 ± 8.68	12.30 ± 8.76
S3 3L-ROI	12.85 ± 9.15	12.89 ± 9.12	12.82 ± 9.10
S4a 3L-ROI	12.61 ± 9.00	12.91 ± 9.16	12.83 ± 8.92
S4b 3L-ROI	13.42 ± 9.59	13.06 ± 9.33	13.30 ± 9.28
S5 3L-ROI	13.37 ± 9.54	13.40 ± 9.50	13.37 ± 9.30
S7 3L-ROI	13.30 ± 9.45	13.45 ± 9.44	13.31 ± 9.43
S6 3L-ROI	14.54 ± 9.86	14.44 ± 10.01	14.43 ± 9.80
S8 3L-ROI	14.08 ± 9.31	14.31 ± 9.58	14.08 ± 9.31
4S-ROI	13.94 ± 9.39	13.84 ± 9.26	13.46 ± 9.17
4L-ROI	13.52 ± 9.25	13.66 ± 9.40	13.46 ± 9.17
9S-ROI	13.63 ± 9.28	13.67 ± 9.28	13.67 ± 9.33
27S-ROI	13.67 ± 9.33	13.60 ± 9.11	13.31 ± 8.99
9L-ROI	13.12 ± 9.16	13.19 ± 9.16	13.07 ± 9.10
27L-ROI	13.12 ± 9.15	13.14 ± 9.18	13.46 ± 9.07

*PDFFs* Proton density fat fractions, *S-ROI* Small region-of-interest, *L-ROI* Large region-of-interest

### Correlations and correlation comparison

The pathological correlations (PDFF and biopsy) of different sampling strategies and correlation comparisons are listed in Tables 1, 2, 3. The results showed that the sampling strategy of S8 1S-ROI and S8 1L-ROI by Reader 2 had correlation coefficients < 0.8 (*r* = 0.796 and 0.788, respectively), while other sampling strategies had correlation coefficients > 0.8 (*r* = 0.811‒0.904). Only the correlations of S3 3L-ROI were greater than those of all sampling strategies at the whole-liver level (4S-ROI, 4L-ROI, 9S-ROI, 9L-ROI, 27S-ROI, and 27L-ROI), and the differences were significant (*P* < 0.05). All pairwise comparisons between the pathological correlations of the two sampling strategies at the whole-liver level had no significant differences (*P* > 0.05).

### ICC and bland–altman analysis

**Fig. 4:**
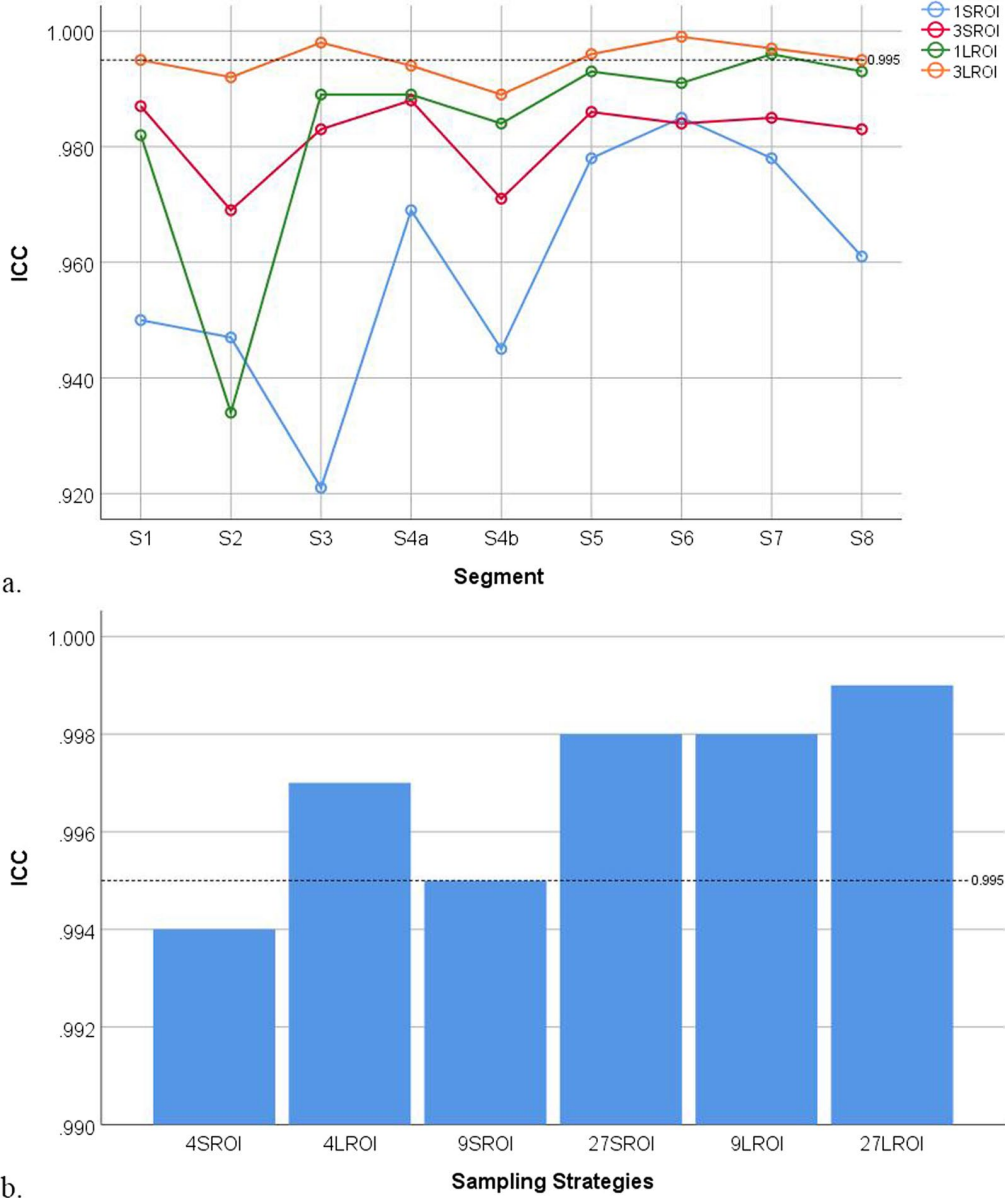
Intra-class correlation coefficient (ICC) of sampling strategies at the segmental and whole-liver levels for inter-reader agreement increased with the number and size of regions-of-interest. **a** The ICC of sampling strategies at the segmental level. **b** The ICC of sampling strategies at the whole-liver level. Each dot or bar represents a particular sampling strategy. The dotted line indicates an ICC of 0.995

**Fig. 5:**
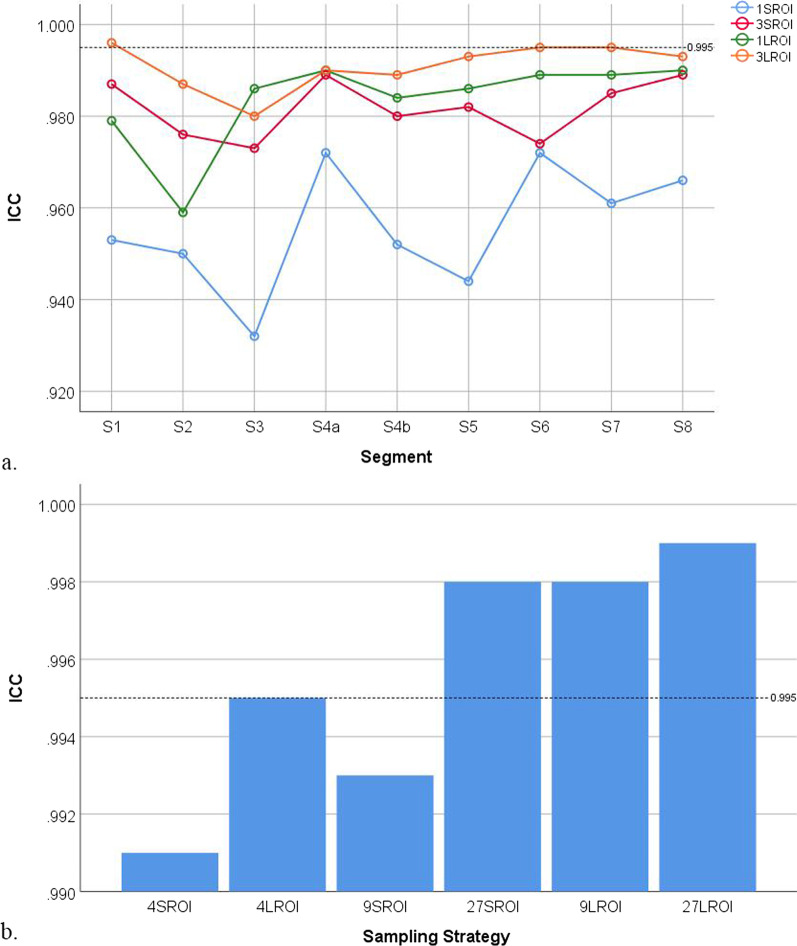
Intra-class correlation coefficient (ICC) of sampling strategies at the segmental and whole-liver levels for intra-reader agreement increased with the number and size of regions-of-interest. **a** The ICC of sampling strategies at the segmental level. **b** The ICC of sampling strategies at the whole-liver level. Each dot or bar represents a particular sampling strategy. The dotted line indicates an ICC of 0.995

**Fig. 6:**
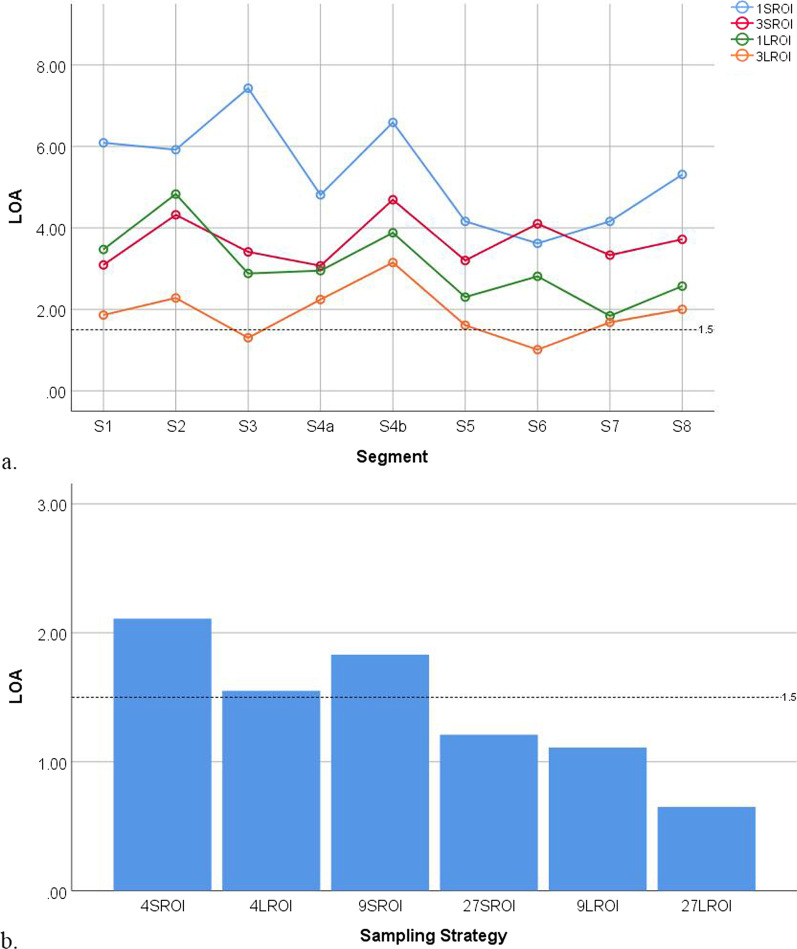
The limits of agreement (LOA) of sampling strategies at the segmental and whole-liver levels for inter-reader agreement decreased with the number and size of regions-of-interest. **a** The LOA of sampling strategies at the segmental level. **b** The LOA of sampling strategies at the whole-liver level. Each dot or bar represents a particular sampling strategy. The dotted line indicates an LOA of 1.5%

**Fig. 7:**
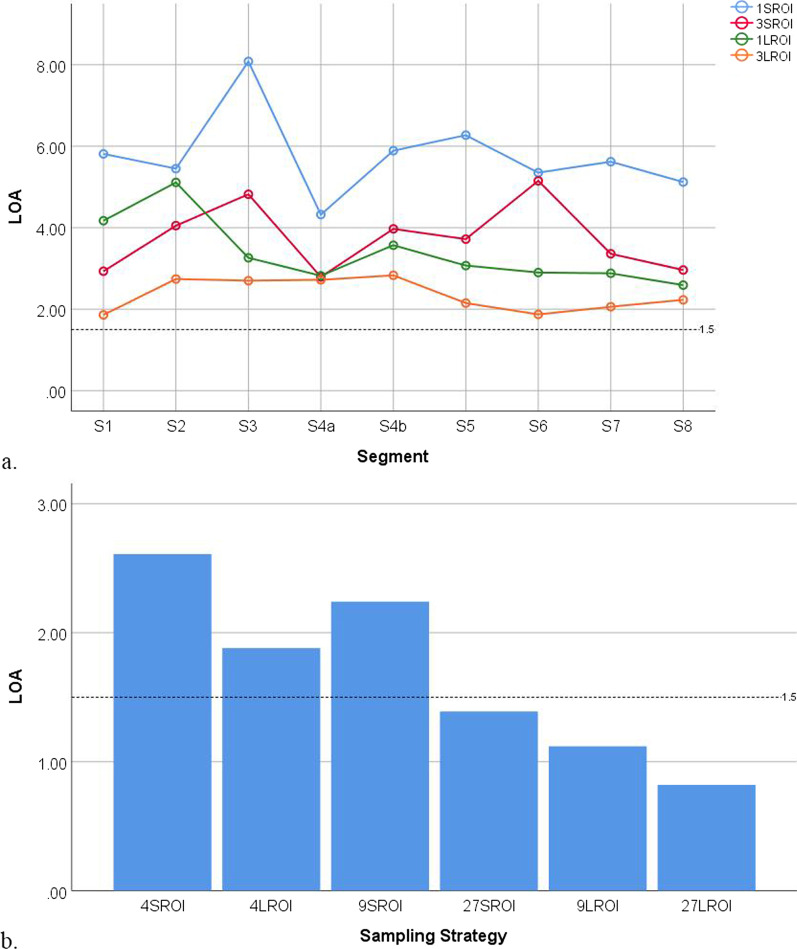
The limits of agreement (LOA) of sampling strategies at the segmental and whole-liver levels for intra-reader agreement decreased with the number and size of regions-of-interest. **a** The LOA of sampling strategies at the segmental level. **b** The LOA of sampling strategies at the whole-liver level. Each dot or bar represents a particular sampling strategy. The dotted line indicates an LOA of 1.5%

ICC and Bland–Altman analysis of intra- and inter-reader agreements using different ROI sampling strategies are summarised in Tables 6 and 7. The results showed a general trend of increasing ICC and decreasing LOA bounds (closer intra- and inter-reader agreements) as the number and size of ROIs increased at both segmental and whole-liver levels (Figs. 4, 5, 6, 7).

**Table 6:** Intraclass coefficients (ICCs) of different sampling strategies

	Reader 1, 1st time vs. Reader 1, 2nd time				Reader 1, 1st time vs. Reader 2			
Sampling strategies at segmental level (S1‒S8)								
	Mean ICC	Highest ICC	Lowest ICC	Proportion with ICC > 0.995	Mean ICC	Highest ICC	Lowest ICC	Proportion with ICC > 0.995
1S-ROI	0.959	0.985	0.932	0%	0.956	0.972	0.932	0%
3S-ROI	0.982	0.988	0.973	0%	0.982	0.989	0.973	0%
1L-ROI	0.983	0.996	0.959	11% (1/9)	0.984	0.990	0.959	0%
3L-ROI	0.995	0.999	0.980	67% (6/9)	0.991	0.996	0.980	33% (3/9)

Sampling strategies at whole-liver level								
	ICC				ICC			
4S-ROI	0.994				0.991			
4L-ROI	0.997				0.995			
9S-ROI	0.995				0.993			
27S-ROI	0.998				0.998			
9L-ROI	0.998				0.998			
27L-ROI	0.999				0.999			

*S-ROI* Small region-of-interest, *L-ROI* Large region-of-interest

**Table 7:** Limits of agreement (LOA) of all different sampling strategies

	Reader 1, 1^st^ time vs. Reader 1, 2^nd^ time				Reader 1, 1^st^ time vs. Reader 2			
Sampling strategies at segmental level (S1‒S8)								
	Mean LOA	Maximum LOA	Minimum LOA	Proportion with LOA < 1.5%	Mean LOA	Maximum LOA	Minimum LOA	Proportion with LOA < 1.5%
1S-ROI	5.34	7.43	3.62	0%	5.77	8.08	4.32	0%
3S-ROI	3.66	4.69	3.07	0%	3.75	5.15	2.79	0%
1L-ROI	3.06	4.83	1.84	0%	3.37	5.11	2.59	0%
3L-ROI	1.90	3.15	1.01	22% (2/9)	2.35	2.83	1.86	0%

Sampling strategies at whole-liver level								
	LOA				LOA			
4S-ROI	2.11				2.61			
4L-ROI	1.55				1.88			
9S-ROI	1.83				2.24			
27S-ROI	1.21				1.39			
9L-ROI	1.11				1.12			
27L-ROI	0.65				0.82			

LOA refers to the highest absolute value of either side of the bound

*S-ROI* Small region-of-interest, *L-ROI* large region-of-interest

For inter-reader agreement, the sampling strategies at the segmental level for S1 3L-ROI, S3 3L-ROI, S5 3L-ROI, S6 3L-ROI, S7 3L-ROI, and S8 3L-ROI had ICCs > 0.995. The sampling strategies at the segmental level for S3 3L-ROI and S6 3L-ROI had LOAs < 1.5%. The sampling strategies at the whole-liver level for 4L-ROI, 9S-ROI, 27S-ROI, 9L-ROI, and 27L-ROI had ICCs > 0.995, while the sampling strategies at the whole-liver level for 27S-ROI, 9L-ROI, and 27L-ROI had LOAs < 1.5%. The sampling strategy for 27L-ROI at the whole-liver level had the largest ICC and narrowest LOA.

For intra-reader agreement, the sampling strategies at the segmental level for S1 3L-ROI, S6 3L-ROI, and S7 3L-ROI had ICCs > 0.995. None of the sampling strategies at the segmental level had LOAs < 1.5%. The sampling strategies at the whole-liver level for 4L-ROI, 27S-ROI, 9L-ROI, and 27L-ROI had ICCs > 0.995, while the sampling strategies at the whole-liver level for 27S-ROI, 9L-ROI, and 27L-ROI had LOAs < 1.5%. The sampling strategy for 27L-ROI had the largest ICC and narrowest LOA.

### Time-burden

The time measurement data are summarised in Table 8. Overall, the time-burden increased with the increase in the size and number of ROIs.

**Table 8:** Time-burden(s) of all region-of-interest strategy measured by Reader 1

	Time-burden
S1 1S-ROI	5.06 ± 1.04
S2 1S-ROI	4.76 ± 0.77
S3 1S-ROI	5.02 ± 0.91
S4a 1S-ROI	4.90 ± 0.89
S4b 1S-ROI	5.14 ± 0.67
S5 1S-ROI	4.88 ± 0.82
S6 1S-ROI	4.82 ± 0.90
S7 1S-ROI	5.10 ± 1.08
S8 1S-ROI	4.68 ± 0.82
S1 3S-ROI	16.10 ± 1.47
S2 3S-ROI	15.42 ± 1.43
S3 3S-ROI	16.20 ± 1.78
S4a 3S-ROI	15.42 ± 1.57
S4b 3S-ROI	15.86 ± 1.03
S5 3S-ROI	15.72 ± 1.63
S6 3S-ROI	15.72 ± 1.49
S7 3S-ROI	16.04 ± 1.73
S8 3S-ROI	15.26 ± 1.40
S1 1L-ROI	6.16 ± 1.81
S2 1L-ROI	5.04 ± 1.01
S3 1L-ROI	5.12 ± 1.22
Sa4 1L-ROI	5.00 ± 0.88
S4b 1L-ROI	5.32 ± 0.68
S5 1L-ROI	5.08 ± 0.99
S6 1L-ROI	5.20 ± 0.97
S7 1L-ROI	5.62 ± 1.43
S8 1L-ROI	4.72 ± 0.97
S1 3L-ROI	18.66 ± 2.59
S2 3L-ROI	16.32 ± 1.43
S3 3L-ROI	16.86 ± 2.26
S4a 3L-ROI	15.68 ± 1.10
S4b 3L-ROI	16.12 ± 0.98
S5 3L-ROI	16.02 ± 1.87
S7 3L-ROI	16.16 ± 1.75
S6 3L-ROI	16.60 ± 1.78
S8 3L-ROI	15.64 ± 1.66
4S-ROI	19.78 ± 3.04
4L-ROI	22.78 ± 2.80
9S-ROI	44.06 ± 3.12
27S-ROI	141.45 ± 5.04
9L-ROI	46.54 ± 3.54
27L-ROI	147.84 ± 6.23

*S-ROI* Small region-of-interest, *L-ROI* Large region-of-interest

The size of ROI had little impact on time-burden. The change from small ROIs to large ROIs increased the measurement time by 6% (0.33 s) for the 1-ROI sampling strategy at the segmental level, by 4% (0.71 s) for the 3-ROI sampling strategy at the segmental level, by 13% (3.00 s) for the 4-ROI sampling strategy at the whole-liver level, by 6% (2.98 s) for the 9-ROI sampling strategy at the whole-liver level, and by 4% (6.39 s) for the 27-ROI sampling strategy at the whole-liver level.

The number of ROIs had a marked impact on time-burden. For the small ROI sampling strategy at the segmental level, the measurement time increased by 69% (10.82 s) when 1-ROI was changed to 3-ROI. For the large ROI sampling strategy at the segmental level, the measurement time increased by 68% (11.20 s) when 1-ROI changed to 3-ROI. Using the 4S-ROI sampling strategy at the whole-liver level increased the measurement time by 21% (4.06 s) as compared with the 3S-ROI sampling strategy at the segmental level. Using the 4L-ROI sampling strategy at the whole-liver level increased the measurement time by 28% (6.35 s) compared with the 3L-ROI sampling strategy at the segmental level. For the small ROI sampling strategy at the whole-liver level, the measurement time increased by 55% (24.28 s) when 4-ROI changed to 9-ROI and by 69% (97.39 s) when 9-ROI changed to 27-ROI. For the large ROI sampling strategy at the whole-liver level, the measurement time increased by 52% (24.26 s) when 4-ROI changed to 9-ROI and by 68% (100.80 s) when 9-ROI changed to 27-ROI.

## Discussion

The ROI sampling strategy in MRI-PDFF may cause marked variability. To improve accuracy and effective PDFF measurement, we evaluated the pathological correlation, intra- and inter-reader agreement, and time-burden of different sampling strategies for MRI-PDFF, with variation in ROI size, location, and number. We found that whole-liver level sampling strategies of 27S-ROI, 9L-ROI, and 27L-ROI provided the best intra-reader agreement and inter-reader agreement. Correlations when using S3 3L-ROI on the puncture site segment were significantly greater than those for all sampling strategies at the whole-liver level. Time-burden increased the most (by 100.80 s) when 9L-ROI strategy was changed to 27L-ROI strategy.

Fat distribution in the liver may not be uniformly distributed [22–24, 36, 37], for numerous reasons. It is hypothesised that adiposis may be related to insulin, because insulin can stimulate the conversion of glucose to fatty acids. Due to the different concentrations of insulin in the "third liver inflow", which are some aberrant small veins (e.g., the gastric and cystic veins) that directly enter the liver, the fat content in specific areas of the liver can be increased or decreased [36, 37]. In addition, the mesenteric vein contains more nutrients, and the blood from the mesenteric vein is preferentially shunted to the right lobe through the right portal vein, while the blood of the splenic vein, with less nutrients, is preferentially shunted to the left lobe through the left portal vein, thereby resulting in more triglyceride deposition in the right lobe [22, 23]. For these reasons, an abnormal distribution may be missed by liver biopsy. Therefore, noninvasive imaging methods, such as MRI-PDFF, which can cover the whole liver and which have excellent diagnostic value, could be preferable for evaluating the fat distribution and longitudinal changes therein. Although MRI-PDFF, as a non-invasive quantitative imaging biomarker, has been proven to have excellent repeatability and reproducibility across different field strengths, manufacturers, and reconstruction approaches [38, 39], it is inevitable that the use of different sampling strategies by radiologists would result in varying PDFFs. Owing to this lack of standardisation in the sampling strategy used, further variability is introduced, which affects the widespread use of MRI-PDFF as a reproducible quantitative imaging biomarker.

Campo et al. [28] measured two ROIs (left and right liver lobes), four ROIs (anterior, posterior, medial, and lateral liver segments), and nine ROIs (nine Couinaud segments) with sizes of 1 cm^2^, 4 cm^2^, and large fit, respectively. They suggested that multiple large ROIs should be used to sample liver PDFF. Hooker et al. [29] performed a secondary cross-sectional and longitudinal analysis of MRI-PDFF data. PDFFs were measured by placing one primary and two additional ROIs in each segment. They found that the 27-ROI and 9-ROI PDFF measurements had ICCs of 0.998 and 0.997, respectively, and that the ICC for primary-ROI, single-segment PDFF measurements ranged from 0.957 to 0.990, depending on the segment. Our results support the above research. In our study, two readers placed different numbers of ROIs of varying sizes in each Couinaud segment to obtain PDFF, using different measurement methods, after which the intra-reader and inter-reader agreements were analysed. For a sampling strategy at the segmental level, the 1S-ROI strategy had the smallest mean ICC and the widest mean LOA, while the 3L-ROI strategy had the largest mean ICC and the narrowest mean LOA. At the segmental level, the 4S-ROI strategy had the smallest ICC and the widest LOA, while the 27L-ROI strategy had the largest ICC and the narrowest LOA. Regardless of intra-reader or inter-reader agreement, 27S-ROI, 9L-ROI, and 27L-ROI strategies at the whole-liver level had ICCs > 0.995 (0.998–0.999) and LOAs < 1.5% (0.56%–1.39%). These results also demonstrated that multiple larger ROIs can increase agreement at both the segmental and whole-liver levels. Hong et al. [20, 21] suggested using a balanced 4-ROI strategy (radius: 1 cm) to measure PDFF. In one previous study [20], all balanced 4-ROI strategies yielded an LOA < 1.5%. In another study [21], all balanced 4-ROI strategies achieved inter- and intra-examination ICCs > 0.998, 92% achieved an intra-examination repeatability coefficient (RC) < 1%, 83% achieved an inter-examination RC < 1%, and all achieved a mean absolute error < 1%. The methods of 4-ROI strategies in our study are similar to those of the balanced 4-ROI strategy. We evaluated the impact of the ROI size of this balanced 4-ROI strategy on reader agreements. We found that the 4S-ROI strategy and the 4L-ROI strategy had LOAs > 1.5%, which do not reach the threshold of LOA < 1.5%. However, the 4L-ROI strategy exceeded the threshold at ICC > 0.995. This suggests that, if PDFF is measured with a balanced strategy, more large ROIs may be required (e.g., 3 ROIs per lobe). The result may have been affected by the small sample size of this study.

In this study, we not only evaluated the reader agreements for some commonly used different sampling strategies at the whole-liver level, but also studied the influence of the number and size of ROIs at the segmental level on the reader agreements, to provide a reference for the determination of a clinical liver PDFF measurement scheme. Idilman et al. [17] previously placed a 4-cm^2^ ROI in segments V and VI and a 2-cm^2^ ROI in other segments to obtain the average PDFF of the liver. They found that the PDFF was closely related to pathological hepatic steatosis (*r* = 0.788–0.904).

Although MRI-PDFF has been proven to be a good indicator of the degree of hepatic steatosis, there is inhomogeneity of fat distribution, resulting in sampling error in liver biopsy [40]. We suspect that placement of ROIs in different regions of the liver may affect the correlation between PDFF and pathology. Liver biopsy is the gold standard for the diagnosis of MAFLD/NAFLD and evaluation of longitudinal liver histological changes [40–45]. For obese and bariatric surgery patients, questions about appropriate biopsy techniques and locations often arise because of the high prevalence of MAFLD/NAFLD and opportunity to perform liver biopsy. Since the criteria for the best selection of MAFLD/NAFLD biopsy technology and puncture location still need to be established [46], it may be necessary to investigate whether the ROI location should be consistent with the location of the liver puncture site.

According to our knowledge, no previous study has reported whether there are differences in the correlation of liver PDFF measured by different sampling strategies with biopsy as the gold standard. An important and unique advantage of the present study is that we used liver biopsy as the standard to compare the pathological correlations (PDFF and biopsy). Although all sampling strategies at the whole-liver level had high correlation coefficients (*r* = 0.864–0.875), the puncture site segment (S3) 3L-ROI sampling strategy yielded the highest correlation coefficient (*r* = 0.895–0.904) in all three measurements. In addition, the correlation of S3 3L-ROI was higher than the correlations of all sampling strategies at the whole-liver level in all three measurements, with statistically significant differences. The reason for this may be that multiple large ROIs placed on S3 may be more suitable for obtaining the PDFF of the liver tissue located at or around the puncture site, offsetting the influence of the sampling error.

Bonekamp et al. [22] used MRI to evaluate the spatial distribution and variability of liver fat in patients without NAFLD. In that study, PDFF in the left lobe was lower than that in the right lobe. Chaim et al. [47] found that the changes in fat content in different liver areas after weight loss surgery in obese patients were different, with right lobe segments having higher PDFF at baseline and showing more rapid reduction in liver fat content. PDFF measured at the whole-liver level may not accurately reflect the histological changes of liver biopsy at the puncture site. For such cases, the sampling strategy of using at least three large ROIs in the puncture site segment may be a reasonable choice, as PDFF measured by this sampling strategy can accurately reflect liver biopsy.

Our study recorded the measurement time-burden in an attempt to provide a compromise between measurement performance of different sampling strategies and the time-burden of liver PDFF. Our results were similar to those of Campo et al. [20], who found that an increase in the number and size of ROIs can increase the time-burden for the reader to obtain the ROI-based liver PDFF artificial measurements. Notably, the increase in the number of ROIs had more effect on time-burden than did the increase in ROI size. The reasons for this result are obvious: A larger ROI makes the reader spend more time observing other structures, such as large blood vessels and bile ducts, to avoid the influence of these anatomical structures, and an increase in the number of ROIs requires the reader to spend relatively more time looking for the corresponding level and location to draw and input data. Therefore, although the sampling strategy of 27L-ROI demonstrates the best consistency, it is the most time-consuming sampling strategy.

Based on the comprehensive consideration of reader agreement, correlation, and trade-off of time-burden, we suggest the 9L-ROI sampling strategy, with an analysis time of approximately 45 s, at the whole-liver level, for hepatic PDFF measurement without liver puncture biopsy as the gold standard and for hepatic PDFF in day-to-day analysis. For hepatic PDFF with liver puncture biopsy as the gold standard, 3L-ROI sampling strategy, with an analysis time of approximately 15 s, at the puncture point segment is suggested.

This study had some limitations. First, this was a single-centre study of adult obese patients in northern China, which may limit the universality of our results. Second, liver biopsy was only obtained at S3 in this study, and an uneven fat distribution in the liver of patients may have affected the results. In future, the corresponding liver biopsy may be used to evaluate and analyse PDFF in different liver lobes or even liver segments, to verify the results of this study. Previous studies have demonstrated the feasibility of whole-liver ROIs and automated MRI liver segmentation to measure PDFF [48–50]. The above methods were not evaluated in this study because such methods cannot avoid anatomical structures, such as large blood vessels, in the liver, which may affect the MRI-PDFF. In addition, no fixed slices were used for ROI measurements in this study, which may have led to variation of PDFF and time-burden.

## Conclusions

Our study showed that PDFF measured by the sampling strategy of 3L-ROI on the puncture site segment can most accurately reflect the liver biopsy. Intra-reader and inter-reader agreements are improved when multiple large ROIs are used to measure PDFF. In addition, the number of ROI has a greater impact on the time-burden than does the size of ROIs. For hepatic PDFF measurement without liver puncture biopsy as the gold standard and for hepatic PDFF day-to-day analysis, 9L-ROI sampling strategy, with an analysis time of approximately 45 s, at the whole-liver level is suggested. For hepatic PDFF with liver puncture biopsy as the gold standard, 3L-ROI sampling strategy, with an analysis time of approximately 15 s, at the puncture point segment is suggested.

## Data Availability

The data that support the findings of this study are available from the corresponding author, but restrictions apply to the availability of these data, which were used under license for the current study, and thus are not publicly available. Data are, however, available from the authors upon reasonable request and with permission of the corresponding author.

## Abbreviations

BMI: Body mass index
ICC: Intra-class correlation coefficients
MRI: Magnetic resonance imaging
MAFLD: Metabolic-associated fatty liver disease
NAFLD: Nonalcoholic fatty liver disease
NASH: Nonalcoholic steatohepatitis
PACS: Picture archiving and communication system
PDFF: Proton density fat fraction
ROI: Region-of-interest
